# Connexins and Nitric Oxide Inside and Outside Mitochondria: Significance for Cardiac Protection and Adaptation

**DOI:** 10.3389/fphys.2018.00479

**Published:** 2018-05-16

**Authors:** Maria Shvedova, Yana Anfinogenova, Sergey V. Popov, Dmitriy N. Atochin

**Affiliations:** ^1^Cardiovascular Research Center and Cardiology Division, Massachusetts General Hospital, Harvard Medical School, Charlestown, MA, United States; ^2^Cardiology Research Institute, Tomsk National Research Medical Center, Russian Academy of Sciences, Tomsk, Russia; ^3^RASA Center, National Research Tomsk Polytechnic University, Tomsk, Russia

**Keywords:** mitohodria, connexins, nitric oxide synthase, ischemia, pre-conditioning

## Abstract

Irreversible myocardial damage happens in the presence of prolonged and severe ischemia. Several phenomena protect the heart against myocardial infarction and other adverse outcomes of ischemia and reperfusion (IR), namely: hibernation related to stunned myocardium, ischemic preconditioning (IPC), ischemic post-conditioning, and their pharmacological surrogates. Ischemic preconditioning consists in the induction of a brief IR to reduce damage of the tissue caused by prolonged and severe ischemia. Nitric oxide (NO) signaling plays an essential role in IPC. Nitric oxide-sensitive guanylate cyclase/cyclic guanosine-3′,5′-monophosphate (cGMP)-dependent protein kinase type I-signaling pathway protects against the IR injury during myocardial infarction. Mitochondrial ATP-sensitive and Ca^2+^-activated K^+^ channels are involved in NO-mediated signaling in IPC. Independently of the cGMP-mediated induction of NO production, *S*-nitrosation represents a regulatory molecular mechanism similar to phosphorylation and is essential for IPC. Unlike conditioning phenomena, the mechanistic basis of myocardial stunning and hibernation remains poorly understood. In this review article, we hypothesize that the disruption of electrical syncytium of the myocardium may underly myocardial stunning and hibernation. Considering that the connexins are the building blocks of gap junctions which represent primary structural basis of electrical syncytium, we discuss data on the involvement of connexins into myocardial conditioning, stunning, and hibernation. We also show how NO-mediated signaling is involved in myocardial stunning and hibernation. Connexins represent an essential element of adaptation phenomena of the heart at the level of both the cardio- myocytes and the mitochondria. Nitric oxide targets mitochondrial connexins which may affect electrical syncytium continuum in the heart. Mitochondrial connexins may play an essential role in NO-dependent mechanisms of myocardial adaptation to ischemia.

## Introduction

Several phenomena protect the heart against myocardial infarction and other adverse outcomes of ischemia and reperfusion (IR) (Skyschally et al., 2008). They include hibernation related to stunned myocardium (Heusch et al., 2005; Depre and Vatner, 2007), ischemic preconditioning (IPC) (Murry et al., 1986), delayed or second-window IPC caused by transient adenosine A1 receptor activation (Marber et al., 1993), ischemic post-conditioning (Zhao et al., 2003), and their pharmacological surrogates (Heusch et al., 2008).

Myocardial recovery usually occurs with a delay when ischemia is moderately severe. This reversible contractile left ventricular dysfunction is recognized as myocardial stunning, a condition which persists for some time after reperfusion (Kudej and Vatner, 2003). There is lack of knowledge on exact causes of stunning. Probable mechanisms include oxidative stress and alteration of calcium homeostasis during reperfusion. Prolonged but less severe ischemia leads to a reversible chronic left ventricular dysfunction known as hibernating myocardium (Ferrari et al., 1996). Hibernating myocardium due to ischemia is named so because myocardial blood flow is chronically reduced similarly to downregulated function and blood flow in animals during true hibernation. The myocardial blood flow is maintained in true mammalian hibernation likely due to a nitric oxide (NO) mechanism (Kudej and Vatner, 2003). Downregulation of myocyte mitochondria (McDiarmid et al., 2017) and altered expression of plasma membrane Cx43 (Kaprielian et al., 1998) are present in hibernating myocardium. However, the intrinsic mechanisms of ischemia-induced myocardial hibernation remain to be elucidated.

Ischemic preconditioning consists in the induction of a brief IR to reduce damage of the myocardium triggered by severe ischemia (Cohen et al., 2000). Remote ischemic conditioning (RIC) is another therapeutic approach where conditioning algorithms are applied to a remote intact organ which confers a global protective phenotype and enables the heart and other organs to resist IR injury. In RIC, neural and humoral signals from the remote organ are transferred to the ischemic heart (Brandenburger et al., 2014; Lotfollahi et al., 2016). Beneficial actions of IPC and ischemic post-conditioning occur primarily in the cardiomyocytes, rather than in smooth muscle cells (Frankenreiter et al., 2017, 2018). Myocardial ischemic conditioning, stunning, and myocardial hibernation are the conditions of adaptation to ischemia activating endogenous mechanisms of cell survival. These states switch gene and protein expression sustaining cardiac cell survival during oxygen deprivation and reperfusion (Rashed and Depre, 2006).

In this paper, we review current knowledge on the role of connexins and NO inside and outside mitochondria in myocardial conditioning phenomena, stunning, and hibernation.

## Inside Mitochondria

### Nitric Oxide Signaling and Cardioprotection

During acute IR injury, activation of the autacoid receptors on the cardiomyocyte plasma membrane recruits endothelial NO synthase (NOS, eNOS) and NO among various signaling pathways protecting mitochondria (Yellon and Downey, 2003; Weerateerangkul et al., 2011). Autacoids such as opioids and bradykinin are released from the heart during IPC and act through the corresponding Gi-coupled receptors (Downey et al., 2008). Upon binding, the receptors activate phosphatidylinositol 3-kinase (PI3-kinase) which upregulates Akt through phosphoinositide-dependent protein kinase. PI3-kinase activates eNOS through extracellular signal regulated kinase (ERK). The generated NO activates soluble guanylate cyclase (GC) producing cyclic guanosine-3′,5′-monophosphate (cGMP) that causes protein kinase G (PKG) upregulation (Downey et al., 2008).

Nitric oxide signaling is involved in the molecular basis of cardioprotection inside and outside mitochondria (Heusch, 2011, 2015). In the mitochondria, it contributes to cardioprotection in IPC (Nadtochiy et al., 2009), which is one of the most successful strategies in attenuation of mitochondrial damage after IR (Totzeck et al., 2017). NO signaling is also a central event in the delayed IPC (Murillo et al., 2011). NO-stimulated mitochondrial biogenesis (Nisoli et al., 2003) results in remodeling of the structure and/or function of the mitochondrion, one of the downstream events of IPC (McLeod et al., 2005). In IPC, eNOS activation occurs upon an upregulation of kinase pathways that minimize apoptosis and tissue damage. In remote IPC, phosphorylation of eNOS and Akt is involved in the reduction of the infarct size. These phenomena act as triggers of remote IPC (Donato et al., 2016).

Nitric oxide-activated PKG transmits the cardioprotective signal from the cytosol to the mitochondria. In the isolated mitochondria, an application of exogenous PKG and cGMP is associated with the opening of mitochondrial KATP channels expressed in the inner mitochondrial membrane. K^+^ influx causes mitochondrial swelling associated with the production of sub-lethal reactive oxygen species (ROS) involved in IPC (Lebuffe et al., 2003; Oldenburg et al., 2004; Qin et al., 2004; Costa et al., 2005; Cuong et al., 2005, 2006; Ljubkovic et al., 2007; Weerateerangkul et al., 2011; Donato et al., 2016) and reperfusion (Downey et al., 2008). These radicals activate PKC through redox signaling which promotes cardioprotection for up to 2 h. The receptors of autacoid adenosine also activate PI3-kinase and can directly affect PKC thus bypassing the mitochondrial pathway involving ROS. Adenosine receptors regulate the survival kinases, Akt and ERK phosphorylating glycogen synthase kinase-3 beta preventing formation of mitochondrial permeability transition pores (Downey et al., 2008). Opening of the mitochondrial KATP channel and the inhibition of mitochondrial permeability transition pore opening are the mechanisms by which mitochondria protect the heart against IR injury (Weerateerangkul et al., 2011). Mitochondrial KATP opening induced by PKG, phorbol ester, or diazoxide is independent of ROS. A positive feedback loop can maintain mitochondrial KATP channel open after cessation of stimulus providing memory effect of preconditioning (Costa and Garlid, 2008).

Independently of mitochondrial KATP activity, exogenous NO inhibits mitochondrial permeability transition pore opening via activation of PKCε_2_ (Costa and Garlid, 2008). This mechanism may be modulated. For example, intake of acrolein, a toxic and irritant substance present in tobacco smoke, worsens ischemic injury of the myocardium as it blocks NO-induced PKCε signaling and cardioprotection (Wang et al., 2008).

Mitochondrial voltage-gated and Ca^2+^-activated K^+^ channels of the ‘big potassium’ (BK)-type are also involved in NO-dependent regulation through GC/cGMP/cGMP-dependent protein kinase type I (cGKI)-signaling pathway (Frankenreiter et al., 2017). Upregulation of cGMP/cGKI signaling stimulates mitochondrial BK channels. In e*x vivo* study, high BK activity contributes to the survival of the myocardium after IR. BK channels seem to allow the cardiac protection triggered by IPC, ischemic post-conditioning, and by different pharmacological agents such as the activators of soluble GC and inhibitors of phosphodiesterase 5 (Frankenreiter et al., 2017). Unlike smooth muscle cells where the damaging effects of the IR do not involve BK, cardiomyocyte-specific deletion of the mitochondrial BK channels makes the heart more prone to IR injury. Mitochondrial BK channels, expressed in the cardiomyocytes therefore are promising targets for preventing acute cardiac damage and adverse outcomes after myocardial infarction (Frankenreiter et al., 2017).

Less mitochondrial permeability transition pore opening occurs when oxidative stress is relieved. IPC attenuates oxidative stress during IR and thus protects the heart (Cadenas et al., 2010). Low NO levels prevent mitochondrial permeability transition pore opening, whereas high NO levels promote its opening and release of cytochrome c (Burwell and Brookes, 2008).

Cytochrome c oxidase is the predominant direct target of NO in the mitochondria. Nanomolar concentrations of NO bind both the reduced and oxidized forms of the cytochrome c oxidase in competition with oxygen leading to the reversible inhibition of oxygen consumption (Cleeter et al., 1994; Poderoso et al., 1998).

The mitochondria, which are damaged during prolonged ischemic episodes, are predominant source of ROS generation which is a trigger of the microvascular dysfunction after IR (Granger and Kvietys, 2017). In the mitochondria, ROS generation after IR is reduced by nitrates (physiological store of NO) which are powerful mediators of cytoprotection (Raat et al., 2009). However, studies on isolated permeabilized cardiomyocytes show that high concentrations of NO donors (0.5–500 mM) impair mitochondrial respiration and induce apoptosis (Ohtani et al., 2012).

An intracellular release of NO triggers a cascade where the production of a limited amount of mitochondrial ROS causes myocardial protection by the mitochondrial permeability transition pore inactivation without onset of oxidative stress (Folino et al., 2013). Vice versa, if the NO release is excessive, the oxidative stress prevails on the protection and causes opening of mitochondrial permeability transition pore (Brown and Borutaite, 2007; Salloum et al., 2007; Folino et al., 2013).

IPC-stimulated nitration reactions result in the formation of nitroalkenes that induce mild mitochondrial uncoupling and protect from IR injury (Nadtochiy et al., 2009). Mitochondrial uncoupling is a process preventing electron transport to drive adenosine triphosphate (ATP) synthesis or to perform other relevant work such as net ion translocation. Mild mitochondrial uncoupling of mitochondria *per se* is cardioprotective. Uncoupling proteins (UCPs) and the adenine nucleotide translocase (ANT) are involved in post-translational modification and cause mild uncoupling of mitochondria during IPC. NO^∗^-derived electrophilic nitrated lipids such as nitro-linoleate may be involved in IPC-induced mitochondrial uncoupling. Nitroalkenes such as nitro-linoleate are endogenously produced in mitochondria of the heart in animal model of IPC (Nadtochiy et al., 2009). Mild mitochondrial uncoupling is inhibited by UCP and ANT antagonists and is activated by synthetic nitro-linoleate which exerts cardioprotective effects against ischemia-reperfusion injury. Biotinylated nitro-linoleate covalently modifies ANT thiols and possibly UCP-2 (Nadtochiy et al., 2009).

Diabetic human myocardium fails to be protected by IPC due to mitochondrial dysfunction (Barua et al., 2011). However, *ex vivo* supply of exogenous NO and suppression of endogenous NO production results in potent cardioprotection in isolated myocardial slices obtained from diabetic patients undergoing elective cardiac surgery (Barua et al., 2011). Hyperglycemia reverses protective effect of IPC, but protection of the myocardium may be rescued by GTP cyclohydrolase 1 overexpression increasing tetrahydrobiopterin and NO concentrations in the myocardium (Ge et al., 2011).

Pharmacological agents can mimic IPC. For example, animal study demonstrated similarities between acute oxytocin pretreatment and IPC in regard to infarct size reduction, antiarrhythmic activity, and metabolic status (Das and Sarkar, 2012). Storage of donor hearts in cardioplegic solutions containing the agents with effects similar to IPC enhances post-reperfusion cardiac function. Glyceryl trinitrate and cariporide activate signaling pathways that favor mitophagy activation and maintain mitochondrial transition pore closure after reperfusion. These pathways may be crucial for functional recovery of the donor heart (Kwan et al., 2015). Administration of nitrite, a dietary constituent and NO oxidation product, shows cardioprotective properties after IR in animals (Kamga Pride et al., 2014). Cardioprotective action of a transient normoxic nitrite treatment depends on the activation of protein kinase A that phosphorylates and inhibits dynamin-related protein 1, the predominant regulator of mitochondrial fission. This promotes mitochondrial fusion and augments mitochondrial membrane potential and superoxide production. ROS, generated during ischemia, target AMP kinase (AMPK) whereas ROS scavenging prevents AMPK activation and inhibits nitrite-mediated protection after hypoxia/reperfusion (Kamga Pride et al., 2014).

The ventricular dysfunction observed in myocardial stunning is associated with a mitochondrial dysfunction that includes partial inactivation of complex I and mitochondrial nitric oxide synthase (mtNOS) activities, oxidative and nitrosative damages and increased H_2_O_2_ and ONOO-production rates (Valdez et al., 2016). Correction of myocardial stunning occurs in patients with cardiac arrest administered with NO donors, which improve cardiac function and cardiac output due to reversible inhibition of mitochondrial complex I and mtNOS additionally to direct vasodilation of coronary arteries (Kruzliak et al., 2014). In the hibernating myocardium, metabolic reprogramming involves HIF affecting mitochondrial bioenergetics associated with NO production (Cadenas et al., 2010).

Metabolic syndrome in patients with myocardial infarction is independent risk factor for major adverse cardiovascular events (Lovic et al., 2018). Mitochondrial dysfunction is involved in the progression of metabolic syndrome and is associated with the changes in eNOS, inducible NOS (iNOS), neuronal NOS (nNOS), and mtNOS signaling (Litvinova et al., 2015).

Targeted genetic models confirm significance of NO-associated signaling in cardiac mitochondria. In comparison with wild-type hearts, isolated iNOS-transgenic hearts subjected to IR display improved mitochondrial function which is associated with better contractile recovery. Increase in iNOS expression in cardiomyocytes is associated with a decrease in ROS after reperfusion, inhibition of mitochondrial swelling, and lower mitochondrial permeability transition (West et al., 2008). In iNOS-transgenic hearts, inhibition of mitochondrial permeability transition with cyclosporin A attenuates reperfusion-induced production of ROS. The opposite effect of this compound is observed in wild type hearts (West et al., 2008). IR upregulates H11 kinase/Hsp22 (Hsp22), a small heat shock protein that provides cardioprotection (equal to IPC) through a NO-dependent pathway in the mitochondria (Laure et al., 2012). The expression of NOS and NO generation are increased in the mitochondria from transgenic mice with cardiac-specific overexpression of Hsp22, which augments the ability of mitochondria to produce NO. In turn, NO stimulates oxidative phosphorylation in normoxic conditions and decreases anoxia-induced oxidative phosphorylation and ROS production. Similar pattern of events is seen in IPC, implying that Hsp22 may be considered a tool for prevention of mitochondrial dysfunction during ischemia (Laure et al., 2012).

#### Cardioprotection and Mitochondrial *S*-nitrosation

Independently of the cGMP-mediated NO production, *S*-nitrosation represents a molecular mechanism of regulation similar to phosphorylation. Mitochondrial *S*-nitrosation can be detected by modified cysteine residue identification using a straightforward and powerful CysTMT(6) switch assay and by mass spectrometry (Murray et al., 2013). Many mitochondrial proteins show increased *S*-nitrosation during cardioprotection (Pagliaro et al., 2011; Sun et al., 2015) and the caveolae transduce acute NO/*S*-nitrosation cardioprotective signaling in IPC hearts (Sun et al., 2015). However, the existence of a mitochondrial source of NO remains a mystery to some extent and should be studied further (Kohr et al., 2014). Data suggest that mitochondria contain a NOS variant and may produce NO via this enzymatic pathway though controversy remains in this regard (Lacza et al., 2004, 2006a,b). NO, generated from non-mitochondrial sources (Katakam et al., 2013, 2016), can affect oxygen consumption and ATP production (Rutkai et al., 2014) in the mitochondria and can form peroxynitrite (Faraci, 2006; Förstermann, 2010). In turn, peroxynitrite is a powerful activator of the mitochondria (Lacza et al., 2003). Protective effects of NO may occur through post-translational modification of the respiratory complexes in the electron transport chain in mitochondria(Sun et al., 2012, 2015). Complex I is especially susceptible to ischemic damage and is a significant target for NO and its metabolites (Nadtochiy et al., 2007). *S*-nitrosation of critical thiols on the complex I results in cytoprotection due to inhibition of the enzyme (Borutaite and Brown, 2006). Hypoxic NO signaling in cardiomyocytes is associated with a post-translational modification of and structural changes in complex I temporarily alleviating ROS burden during reperfusion (Ertracht et al., 2014; Totzeck et al., 2017). The *S*-nitrosation of cysteine 39 on the ND3 moiety of complex I triggers conformational change decreasing its ROS-production activity. Administration of mitochondrial *S*-nitrosating agent reduces the infarct size (Chouchani et al., 2013). *S*-nitrosoglutathione, NO, or *S*-nitroso-2-mercaptopropionyl glycine also shows cardioprotective potential *ex vivo* and *in vivo* due to *S*-nitrosation (Nadtochiy et al., 2007). Mitochondrial *S*-nitrosation by NO is essential for cardioprotection against IR injury (Weerateerangkul et al., 2011; Sun et al., 2015). A protective signaling pathway associated with mitochondrial *S*-nitrosation and complex I inhibition (Murray et al., 2013) may be pharmacologically augmented (Nadtochiy et al., 2007).

### Connexins in Mitochondria

Connexins are present at ventricular gap junctions and also in the mitochondria of the murine, porcine, and human cardiomyocytes (Boengler et al., 2005; García et al., 2018). The presence of connexin 43 (Cx43) in the mitochondria was demonstrated for the first time by (Li et al., 2002). The functions of mitochondrial Cx43 comprise regulation of mitochondrial respiration, potassium fluxes (Heinzel et al., 2005; Peart et al., 2014; Denuc et al., 2016; Boengler and Schulz, 2017), oxygen consumption (Boengler and Schulz, 2017), mitochondrial Ca^2+^ homeostasis (Guo et al., 2017), and the mitochondrial protein import machinery (Rodriguez-Sinovas et al., 2006). Involvement of mitochondrial Cx43 into the regulation of mitochondrial K^+^ flux in the cardiomyocytes occurs through forming hemichannel-like structures (Miro-Casas et al., 2009). Cx43 interacts with several other proteins playing an important role in the mitochondrial function and metabolism. Apoptosis-inducing factor and the beta-subunit of the electron-transfer protein are present in the subsarcolemmal mitochondrial fraction and known to be related with the respiratory chain. These proteins directly interact with mitochondrial Cx43. These new protein-protein interactions in the subsarcolemmal mitochondria may be implicated in the regulation of the mitochondrial redox state (Denuc et al., 2016). There is strong evidence indicating that mitochondrial Cx43 modulates respiratory complex I-associated mitochondrial respiration, ROS production, and ATP synthesis (Rodríguez-Sinovas et al., 2018). These functions modulate mitochondrial and cellular tolerance to reperfusion after prolonged ischemia and are essential for cardioprotective effects of IPC. Mitochondrial Cx43 may constitute a new pharmacological target in patients with ST-segment elevation myocardial infarction (Schulz et al., 2015).

The presence of Cx43 in the inner mitochondrial membrane plays a central role in IPC (Michela et al., 2015) which itself enhances the mitochondrial localization of this protein (Boengler et al., 2005). The levels of mitochondrial Cx43 expression increase fast after only two 5-min cycles of IR (Boengler et al., 2005). This effect is specific to Cx43 and is not replicated in the mitochondria expressing connexin 32 (Ruiz-Meana et al., 2014). Attenuation of mitochondrial Cx43 abolishes cardioprotective effects of IPC (Boengler et al., 2007) but not post-conditioning (Schulz et al., 2007). Mitochondrial Cx43-dependent cardioprotection in response to diazoxide may be related the Cx43-mediated gating of mitochondrial KATP channels (Heinzel et al., 2005). Interestingly, subpopulations of the mitochondria within one cell differ from each other. eNOS, connexin-43, and caveolin-3 are detected in the sarcolemmal mitochondria, but not in the interfibrillar mitochondria, and IPC further significantly increases eNOS/caveolin-3 levels only in this subpopulation (Sun et al., 2015). Cx43 is exclusively localized in subsarcolemmal mitochondria and its carboxy-terminus is directed toward the intermembrane space. Since loss of mitochondrial Cx43 abolishes cardioprotection due to IPC, the signal transduction of IPC is distinct in the subsarcolemmal and interfibrillar mitochondria exerting different functions (Boengler et al., 2009). Compared with interfibrillar mitochondria, subsarcolemmal mitochondria are more sensitive to fibroblast growth factor 2-triggered cardioprotection against Ca^2+^-induced permeability transition. The cardioprotective effect of this growth factor is linked to mitochondrial Cx43 channel-mediated pathway associated with Cx43 phosphorylation by PKCε in the mitochondria (Srisakuldee et al., 2014).

The subsarcolemmal mitochondria from caveolin-3 knockout mouse hearts are void of ability to IPC. Interestingly, this pool of mitochondria lacks IPC-induced enhancement in the levels of *S*-nitrosation and eNOS/caveolin-3 (Sun et al., 2015). The subsarcolemmal mitochondria may be the primary target of sarcolemmal signaling-derived post-translational protein modification (caveolae-derived eNOS/NO/*S*-nitrosylation) which is pivotal for cardioprotection induced by IPC (Sun et al., 2012, 2015).

Cx43 expression interferes with the expression of NOS isoforms. Vice versa, Cx43 gene expression is down-regulated by NO produced by iNOS. In wild-type mice, isolated mitochondria predominantly contain nNOS. In opposite, iNOS expression is increased in mitochondria from Cx43-deficient mice. The production of NO is lower in mitochondria from Cx43-deficient mice compared with that in wild-type. A reduced mitochondrial Cx43 content is associated with a switch of the NOS isoform and the respective mitochondrial rate of NO production (Kirca et al., 2015).

*S*-nitrosation of the mitochondrial Cx43 increases mitochondrial permeability for potassium and results in elevated ROS formation in the heart. The increased *S*-nitrosation of mitochondrial Cx43 by IPC or nitrite administration may link NO and Cx43 in the signal transduction cascade of cardioprotection (Soetkamp et al., 2014). In the context of redox potential regulation in the mitochondria, NO modulates the hemichannels and the properties of gap junction channels by modulating their trafficking, formation, and functional properties (García et al., 2018).

Interleukin-18 (IL-18), regulating mitochondrial function and connexin-based gap-junction turnover in the cardiomyocytes (Li et al., 2016), can link connexins and NO signaling extending pathophysiological implication of connexins beyond their role in the myocardial conditioning. IL-18 is an interferon-γ-inducing factor and a proinflammatory cytokine ubiquitously expressed both in immune and inflammatory cells, and in non-immune cells (Li et al., 2016). In the atrial cardiomyocytes, IL-18 regulation via NO production results in the combined effects on mechano-gated and Ca^2+^ channels (Kazanski et al., 2017).

Physical exercise, known to increase NO bioavailability (Nosarev et al., 2015) in the cardiovascular system and to boost eNOS-dependent mitochondrial biogenesis in the heart (Vettor et al., 2014), significantly benefits cardiovascular system by improving mitochondrial function through restoring Cx43 networks and mitochondrial trans-membrane potential and by preventing excessive mitochondrial fission (Veeranki et al., 2016).

Mitochondrial Cx43 is targeted by several protein kinases (Schulz et al., 2007) and is involved in Cx43-JNK-Bax axis regulating the process of apoptosis (Uzu et al., 2017). Cx43 (Major et al., 2017) including mitochondrial form of this protein (Shan et al., 2015) is involved in the pathogenesis of dilated cardiomyopathy. In a rat model, dephosphorylation of mitochondrial Cx43 at serine 368 occurs due to the suppression of PKCε activity that may represent a novel mechanism of mitochondrial dysfunction in the pathogenesis of cardiac diseases (Shan et al., 2015).

Cx43 is involved in the effects of pharmacological medications. An increase in Cx43 phosphorylation is associated with an improvement of mitochondrial function due to combination treatment with metformin and vildagliptin in animal model on high fat diet. Treated animals show delayed time to ventricular tachycardia/fibrillation onset, reduced arrhythmia score, and lower mortality rate (Apaijai et al., 2014). Anti-inflammatory and antiulcer drug with nootropic effects Carbenoxolone induces swelling of isolated rat liver and brain mitochondria. Carbenoxolone may exert its effect on permeability transition pore acting via Cx43 as well as via mitochondrial outer membrane translocator protein. Opening of Ca^2+^-induced permeability transition pore is promoted through targeting of Cx43 in isolated rat brain mitochondria. When a threshold Ca^2+^ load is achieved, mitochondrial permeability transition opening occurs as seen from mitochondrial membrane potential drop and efflux of accumulated Ca^2+^ from the mitochondrial matrix (Azarashvili et al., 2014). In turn, mitochondrial permeability transition is the end effector of cardioprotection. Phosphodiesterase 5 inhibitors exert cardioprotection against ischemia/reperfusion injury through NO/cGMP signaling eventually leading to phosphorylation of glycogen synthase kinase-3β representing a master switch immediately proximal to mitochondrial permeability transition pore (Das et al., 2015). An increase in Cx43 expression is associated with the effects of caffeic acid phenethyl ester, an active component of propolis exerting antioxidative, anti-inflammatory, antiproliferative, and antineoplastic properties, reduces cell death in cardiomyocytes (Chen et al., 2016). Caffeic acid phenethyl amide is an analog of caffeic acid phenethyl ester with more structural stability in plasma. Caffeic acid phenethyl amide exerts cardioprotective activity in ischemia/reperfusion injury through antioxidant activity and by preserving NO levels. Prolonged treatment with this compound may ameliorate cardiac dysfunction in diabetes (Ho et al., 2014).

Cardiotoxicity of chemotherapeutic agents remains a life-threatening issue. Cardioprotective properties of mitochondrial Cx43 were shown in the study of cardiotoxicity and myocardial dysfunction caused by doxorubicin in the embryonic cardiomyocyte cell line H9c2 (Pecoraro et al., 2015). Pharmacological inhibition of heat shock protein 90 (Hsp90) showed that the mitochondrial Cx43 exerts cardioprotective effects through attenuation of cytosolic and mitochondrial ROS production, mitochondrial Ca^2+^ overload, mitochondrial membrane depolarization, and cytochrome c release (Pecoraro et al., 2015). In glioblastomas treatment, a Hsp90-dependent mitochondrial translocation of Cx30 following radiation and an improved ATP production following the genotoxic stress are involved in radioprotection. Cx30 is considered a potential biomarker and target for therapeutic intervention (Artesi et al., 2015). Hsp90 may be involved in potential interactions of NO signaling and heat shock proteins. Indeed, Hsp90 activates NOS during exercise in the heat thus contributing to the heat loss response of cutaneous vasculature (Fujii et al., 2017).

Cx43 is co-immunoprecipitated with Tom20 (translocase of the outer membrane 20) and with Hsp90 suggesting the interaction with the regular mitochondrial protein import machinery (Rodriguez-Sinovas et al., 2006). The effects of geldanamycin, a blocker of Hsp90-dependent translocation of proteins to the inner mitochondrial membrane through the TOM pathway, on the mitochondrial Cx43 content in the absence or presence of diazoxide and on the protection against infarction demonstrated that Cx43 is transported to the inner mitochondrial membrane through TOM complex-dependednt translocation and that a normal mitochondrial Cx43 content is essential for preconditioning (Rodriguez-Sinovas et al., 2006). NO may potentially be involved in these pathways as there is evidence that Hsp90 regulates the respiration of cultured neonatal cardiomyocytes (cardiac H9c2 cells) through NOS activation (Ilangovan et al., 2004).

## Outside Mitochondria

### Nitric Oxide Signaling

Outside mitochondria, induction of cardioprotective NO signaling may occur through several pathways: the supplementation with NO-donors, the administration of the ’hypoxic-NO donors nitrate and nitrite, and remote ischemic preconditioning enhancing endogenous NO formation (Schulz et al., 2004; Totzeck et al., 2017). Ischemic preconditioning protects heart from IR injury through the eNOS/NO signaling pathway where it increases phosphorylation of eNOS protein and decreases the expression of iNOS (Qian et al., 2015).

NO plays an essential role in the hibernating myocardium. Phosphorylation of eNOS, simulated by an introduction of a phosphomimetic eNOS construct (eNOS S1177D) into chronic ischemic myocardium, induces neovascularization and improves functional reserve of the hibernating myocardium in a pig model of total occlusion of the left anterior descending artery for 28 days (Kupatt et al., 2007). In both human and animal studies, nitroglycerin induces a protective phenotype preventing tissue damage after IR. This effect is similar to IPC and involves similar molecular pathways (Gori et al., 2008). However, it is important that the optimal NO dose is difficult to determine whereas excessive NO levels may be harmful.

Primary receptor of NO is a soluble GC that synthesizes cGMP activating PKG (Ignarro, 2002). eNOS-derived NO acts as an endogenous cardioprotective agent during IPC (Jones et al., 1999) and iNOS mediates the late phase of IPC (Guo et al., 1999). NO-GC signaling in cardiomyocytes plays an important role for the cardioprotective signaling following AMI *in vivo* (Frankenreiter et al., 2018). Cyclooxygenase-2 and iNOS are co-expressed in the hibernating myocardium with nitrotyrosine suggesting that NO and peroxynitrite are produced after ischemia-reperfusion. These mechanisms modulate myocardial contractile function and survival (Baker et al., 2002).

In transgenic mice, a smaller infarct area is seen in the animals that express more eNOS compared with wild type mice (Jones et al., 2004). Cardiomyocyte-specific increase in iNOS expression also results in sustained cardioprotection (West et al., 2008).

### Connexins Outside Mitochondria

At the plasma membrane, connexins form hemichannels and gap junction channels (García et al., 2018). Six connexin subunits build a connexon (or hemichannel) in the plasma membrane. Two hemichannels interact to form a gap junction channel necessary to pass electrical current flow between the cells or to exchange signaling molecules and metabolic substrates. Connexins participate in autocrine and paracrine intercellular communication through these channels. Connexin-based channels are strongly regulated by membrane potential, phosphorylation, pH, redox potential, and divalent cations. The imbalance of this regulation has been linked to many acquired and genetic diseases (García et al., 2018). Pathophysiological alterations in connexins occur in hypertension, hypertrophy, diabetes, hypercholesterolemia, ischemia, post-myocardial infarction remodeling or heart failure (Schulz et al., 2015).

Cx43 is highly expressed in the cardiomyocytes (Michela et al., 2015) and is essential for IPC of the heart (Denuc et al., 2016) where it improves gap junctional communication and action potential propagation. Gap junctions are required for coupling of cardiomyocytes into a syncytium (Leybaert et al., 2017). The coupling mechanisms associated with the intercalated disks comprise electric field coupling, ephaptic coupling, K^+^ accumulation, and capacitive coupling where the capacitive coupling may exert both excitatory and inhibitory effects (Akbari et al., 2014). Spreading electrical excitation occurs from cell to cell in the heart via the low resistance gap junctions. Non-gap junction-mediated ephaptic mechanisms facilitate propagation of action potentials in tandem with gap junction-mediated coupling that maintains cardiac conduction. Perinexal (within 200 nm of the gap junction plaque) sodium channels function as an ephapse underlying cell-to-cell transfer of electrical excitation. Acute interstitial edema can occur during IR. Edema increases intermembrane distance at the perinexus, slow preferential transverse conduction, and contribute to spontaneous arrhythmia (Veeraraghavan et al., 2015). Connexins are involved in slow calcium wave spreading and transfer of survival or death signals in the cardiomyocytes (Michela et al., 2015).

Remote ischemic conditioning is associated with increased Cx43 phosphorylation 2 h after reperfusion in the area at risk (Alburquerque-Béjar et al., 2016). Considering that myocardial Cx43 and its phosphorylation are involved in arrhythmogenesis (Sanchez et al., 2011), RIC-associated cardioprotection could be attributed to the antiarrhythmic effects of Cx43 upregulation. However, improved Cx43-mediated communication cannot explain powerful protection against ventricular arrhythmias 5 min after reperfusion in animals subjected to combination therapy with RIC and metabolic treatment (glucose-insulin-potassium) (Alburquerque-Béjar et al., 2016).

During reperfusion, a temporary reduction of cardiomyocyte coupling protects against necrosis and limits its size. Downregulation of Cx43 or its replacement by less-conductive Cx32 exerts arrhythmogenic effects under normoxia and ischemia-reperfusion without changing baseline electrical properties (Sanchez et al., 2011).

Cx43 is involved in the pathological progression of myocardial edema in stunned myocardium (Yan et al., 2013). Altered patterns of expression of plasma membrane Cx43, responsible for passive conduction of the cardiac action potential, are involved in the pathogenesis of myocardial hibernation in humans. In hibernating myocardium, the size of Cx43 gap junctions at the periphery of the intercalated disk is significantly reduced, and the overall amount of Cx43 per intercalated disk is attenuated, relative to normally perfused and reversibly ischemic segments of the same heart (Kaprielian et al., 1998; Severs et al., 2004). Progressive reduction and disruption of Cx43 gap junctions in the hibernating myocardium result in abnormal impulse propagation leading to a temporary electromechanical dysfunction associated with the hibernation (Kaprielian et al., 1998). Dedifferentiated hibernating cells are present in the border zone of an infarct area and in ventricular or atrial tissue in cardiac diseases (Sharov et al., 1997; Thijssen et al., 2000; Dispersyn et al., 2002; Thomas et al., 2002). These hibernating cells are involved in heterocellular communication based on Cx43 gap junctions between fibroblasts and cardiomyocytes (Driesen et al., 2005).

Connexins are a downstream target for many regulatory pathways. Post-translationally, connexins are modified by nitros(yl)ation, phosphorylation/de-phosphorylation (Schulz et al., 2015), glycosylation, proteolysis, *N*-acetylation, *S*-nitrosylation, ubiquitination, lipidation, hydroxylation, methylation, and deamidation (D’hondt et al., 2013). All these phenomena can modulate channel activity. Knockout and knockin technology as well as pharmacological approaches show that Cx43 is important for protection from cardiac ischemia/reperfusion injuries (Schulz et al., 2015).

Nitric oxide modulates gap junction permeability regulation and expression of connexin isoforms. Though the mechanistic basis of NO-mediated regulation of these channels remains poorly understood, it is known that NO-induced cardioprotection against IR injury may be beyond the cGMP/PKG-dependent pathway in the isolated cardiomyocytes. NO-mediated *S*-nitrosylation significantly affects cardioprotection in IPC that may depend on *S*-nitrosothiol signaling (Xie et al., 2017). Guanylyl cyclase is a heme-containing a1b1 heterodimer (GC1) generating cGMP in the presence of NO. The NO-GC1-cGMP signaling pathway attenuates contractility and protects cardiomyocyte against remodeling in an angiotensin II hypertrophy model. The b1 subunit of GC1 localizes with Cx43 at the intercalated disk. In the ventricular myocytes, GC1 modulates Cx43 location which affects gap junction function, and, in part, protects from electrical dysfunction. Disruption of the NO-cGMP pathway cause abnormal Cx43 phosphorylation and cardiac electrical abnormalities. Therefore, NO/Cx43 signaling may protect against stress-induced arrhythmias (Crassous et al., 2017). In IPC-induced cardioprotection, an increase in NO production is associated with PKC-ε translocation, Cx43 phosphorylation, and chemical gap junction uncoupling (Rong et al., 2016). NO is involved in the mechanistic basis of IPC failure in heterozygous Cx43 deficient mice and aged wild-type mice (Dawn and Bolli, 2002; Boengler et al., 2007).

Connexins are the key elements conferring myocardium the property of electrical syncytium with extensive intercellular communication of electrical synapse type. Recent studies (Freeman et al., 2014) suggest that several synapses from a single neurite or from the processes of different neurons innervate each cardiomyocyte. This cardiomyocyte-neuronal mesh involves other cell types such as cardiac fibroblasts and endothelial cells (Driesen et al., 2005; Franzoso et al., 2016).

## Conclusion

**FIGURE 1:**
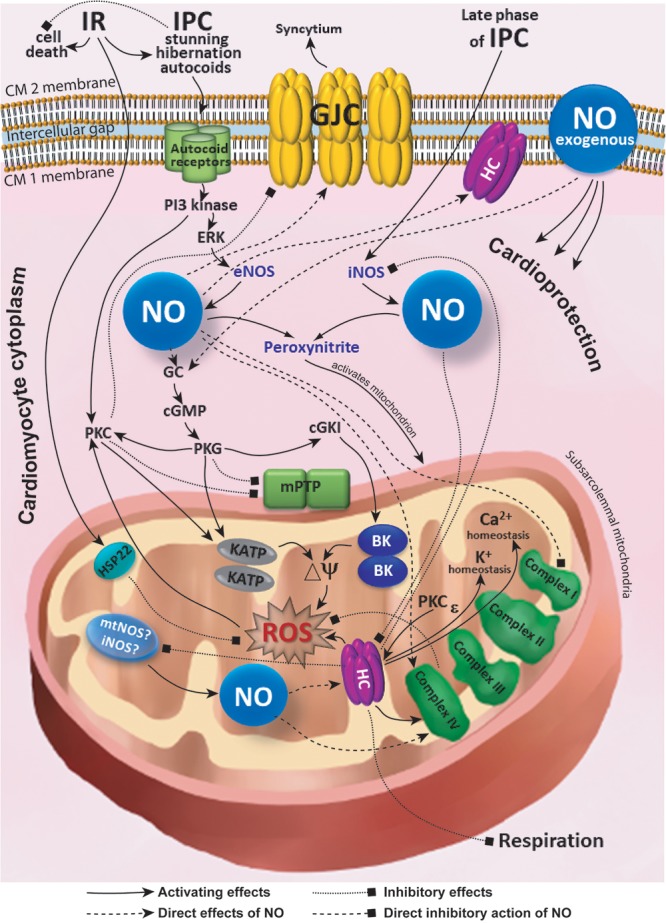
Simplified scheme of interplay between the connexins and NO-medicated signaling inside and outside mitochondria. BK, large-conductance Ca^2+^-activated K^+^ channels; cGKI, cGMP-dependent protein kinase type I; cGMP, cyclic guanosine monophosphate; CM, cardiomyocyte; Cx43, connexin 43; eNOS, endothelial nitric oxide synthase; ERK, extracellular signal regulated kinase; GC, guanylate cyclase; GJC, gap junction channel; HC, hemichannel; HSP22, heat shock protein 22; iNOS, inducible nitric oxide synthase; IPC, ischemic preconditioning; KATP, ATP-sensitive K^+^ channel; mPTP, mitochondrial permeability transition pore; mtNOS, mitochondrial nitric oxide synthase; NO, nitric oxide; PI3, phosphoinositide 3; PKC, protein kinase C; PKG, protein kinase G; ROS, reactive oxygen species; ΔΨ, membrane potential.

Interplay between the connexins and NO-medicated signaling inside and outside mitochondria (**Figure 1**) plays a pivotal role in conditioning phenomena and in cardiac protection. During IR, connexins suppress respiration (and conserve energy) at the level of the mitochondrion and are involved in the modulation of electrical and chemical syncytium at the level of intercellular interaction. Depending on its source (eNOS, iNOS, or mtNOS), place of action (inside or outside the mitochondrion), and adaptive phenomena (the phase and type of IPC), NO directly or indirectly upregulates or downregulates gap junctions and hemichannels. These processes exert cardioprotective action and allow the cells to recover. The organization of cardiac tissue may represent a ‘multicellular synapse’ where connexins, involved in hemichannels and gap junction channels, play an essential role and integrate both cells and intracellular organelles into the continuous functional syncytium. Understanding pathophysiology and pharmacology of connexin-based syncytium of the heart and its modulation by NO signaling, IR, and conditioning is promising for solving the challenges of cardioprotection. Connexins could also play an essential role in the myocardial stunning and hibernation. However, current experimental evidence is still sparse in this regard. Future structural and functional studies of the elements of connexin- and NO-dependent signaling inside and outside cardiac mitochondrion are in demand to understand their impact on the myocardial conditioning, stunning and hibernation. Further research in this direction will potentially reveal new cardioprotective mechanisms and promising molecular targets relevant for translation of bench studies to bedside success.

## Author Contributions

MS researched literature, wrote the manuscript, and contributed to discussion. YA wrote the manuscript and contributed to discussion. SP reviewed, edited the manuscript, and contributed to discussion. DA reviewed, edited the manuscript, and contributed to discussion.

## Conflict of Interest Statement

The authors declare that the research was conducted in the absence of any commercial or financial relationships that could be construed as a potential conflict of interest.
